# Comparison of image registration methods for combining laparoscopic video and spectral image data

**DOI:** 10.1038/s41598-022-20816-1

**Published:** 2022-09-30

**Authors:** Hannes Köhler, Annekatrin Pfahl, Yusef Moulla, Madeleine T. Thomaßen, Marianne Maktabi, Ines Gockel, Thomas Neumuth, Andreas Melzer, Claire Chalopin

**Affiliations:** 1 Innovation Center Computer Assisted Surgery (ICCAS), Faculty of Medicine, Leipzig University, 04103 Leipzig, Germany; 2 Department of Visceral, Thoracic, Transplant, and Vascular Surgery, University Hospital of Leipzig, 04103 Leipzig, Germany

**Keywords:** Biomedical engineering, Image processing, Optical spectroscopy, Imaging and sensing, Endoscopy

## Abstract

Laparoscopic procedures can be assisted by intraoperative modalities, such as quantitative perfusion imaging based on fluorescence or hyperspectral data. If these modalities are not available at video frame rate, fast image registration is needed for the visualization in augmented reality. Three feature-based algorithms and one pre-trained deep homography neural network (DH-NN) were tested for single and multi-homography estimation. Fine-tuning was used to bridge the domain gap of the DH-NN for non-rigid registration of laparoscopic images. The methods were validated on two datasets: an open-source record of 750 manually annotated laparoscopic images, presented in this work, and in-vivo data from a novel laparoscopic hyperspectral imaging system. All feature-based single homography methods outperformed the fine-tuned DH-NN in terms of reprojection error, Structural Similarity Index Measure, and processing time. The feature detector and descriptor ORB1000 enabled video-rate registration of laparoscopic images on standard hardware with submillimeter accuracy.

## Introduction

The intraoperative support of laparoscopic procedures with additional information from pre- or intraoperative data has great potential for the improvement of patient safety and the reduction of operative time. Sufficient organ perfusion is essential during minimally invasive surgery (MIS) and a prerequisite for anastomotic healing, therefore intraoperative perfusion imaging is an emerging field. A non-invasive method for intraoperative visualization of perfusion is hyperspectral imaging (HSI). The potential of HSI for a variety of applications, especially surgery, was shown in several clinical studies^1,2^. Spectral imaging with a high spatial and spectral resolution is obtained with push-broom scanning. In this technique, one spatial and the spectral dimension are acquired at once with a spectrograph, while the second spatial dimension is obtained by moving the sample^3^, the imaging system^4^, or the spectrograph inside the housing^5^. Depending on the obtained wavelengths, physiological tissue metrics like oxygenation and hemoglobin or water content can be derived from the HSI data. This information is visualized as a false-color image but is typically not available at video frame rate^6^. To guide the localization of tissue between static false-color images and video during open surgery, both modalities were overlayed in Barberio et al.^7^, provided that the object and imaging system are not moved. A laparoscopic system for simultaneous video and HSI was presented in Köhler et al.^5^, which provides the hardware requirements for video-rate augmentation of static hyperspectral data aimed in this work. During HSI, a frame of the video is saved as the reference image and continuously aligned with the current frame after hyperspectral scanning is finished. The obtained image transformation enables the overlay of static HSI information and the current frame to support intraoperative localization. Depending on the used registration method, movements of the laparoscope and tissue deformations can be taken into account for HSI overlay visualization. This work mainly aims to compensate for motion due to breathing and small movements of the laparoscope after HSI record. In case of large tissue deformation, occlusion, or perspective change, a new HSI record can be acquired in a few seconds. For common working distances during abdominal MIS, registration errors up to 5 mm are clinically acceptable^8^. Visualization of the resulting overlay should be provided at video frame rate and without additional user input.

### Image augmentation with pre-/intraoperative data

Since augmented reality (AR) for image-guided interventions was introduced in neurosurgery, many laparoscopic applications have also been addressed by AR-related research^9^. Abdominal minimally invasive surgery (MIS) is particularly challenging in this context, due to smooth organs that elastically deform, tissue motion caused by breathing or manipulation, surgical instruments in the field of view, non-planar surfaces, and parallax.

For the localization of lesions in soft organs during MIS, preoperative data from CT or MR can be beneficial. The long-term augmentation of laparoscopic images with a 3-D kidney model from CT data was achieved by an initial manual annotation of corresponding points and subsequent feature tracking and recovery with high accuracy in Puerto-Souza et al.^10^. Intraoperative AR-guided MIS of the uterus with preoperative MR data is demonstrated in Collins et al.^11^, using an exploratory video for initial registration and multiple keyframes with manual input to improve organ tracking. These works show impressive results for intraoperative registration with preoperative data. However, these methods are only partially suitable for overlaying intraoperative image data, as manual user input and 3D models are required.

Fluorescence imaging with Indocyanine Green (ICG) can be used to visualize perfusion, but only qualitative information can be provided in real-time. Quantitative perfusion maps are created from a sequence of fluorescence images and require subsequent registration for color video overlay. That was shown by Selka et al.^8^ with feature detection, description, and matching of the reference image and the current frame. To address local organ deformations, a non-rigid Moving Least Squares (MLS) deformation grid, previously described in Schaefer et al.^12^, was used and tested in an animal model. However, image registration was only achieved at 5 frames per second.

### Image feature detection, description, and matching

Most AR methods use image features, that can be automatically detected by a variety of methods benchmarked by Tareen et al.^13^ on non-surgical datasets. Scale-invariant feature transform (SIFT) was found to be the most accurate, while Oriented FAST and Rotated BRIEF (ORB1000) was the fastest feature-detector-descriptor algorithm. A comparison of seven feature detectors on the green channel of standard color videos from brain surgeries showed, that KAZE^14^ was the most robust and provided the highest density of features^15^. For homogenous spatial distribution of features, fast implementations of Adaptive Non-Maximal Suppression (ANMS) have been described^16^. The description of features can be accelerated by the use of BEBLID, a binary descriptor trained with AdaBoost^17^.

After detection and description, feature matching is used to find corresponding points in different images. Puerto-Souza et al.^18^ presented a hierarchical multi-affine (HMA) algorithm, based on k-means clustering of features, to address the difficulties of feature matching in MIS images with non-planar and moving objects. HMA provides accurate results for individual image regions, but many dense features are required to cover the entire image, which increases computation time.

Matched features can be used for fast and long-term tracking of tissue or instruments in laparoscopic scenes^19^. To address the specific challenges of feature tracking in MIS image data, affine-invariant anisotropic regions^20^ and methods for selecting the most suitable algorithm based on the input data at runtime were proposed^21^. Although these methods are promising, they are computationally expensive and continuous feature tracking is not necessary for image registration.

Finally, the corresponding features of two images are required for the estimation of the homography, a 3 × 3 transformation matrix used for image alignment, obtained by Random sample consensus (RANSAC).

### Deep homography estimation (DH-NN)

Recently, deep convolutional neural networks were used for homography estimation (DH-NN) on non-clinical images for the first time^22^. DH-NN methods do not require matched features, which is beneficial in scenes with low texture, challenging light conditions, or clustered features. On the other hand, a lot of image data is needed for training and the processing is computationally expensive. The process for generating synthetic training data for DH-NN by applying random perspective transformations described in DeTone et al.^22^ was transferred to capsule endoscopy and thus used in the clinical context^23^. That method was applied to image sequences and developed further for the continuous generation of synthetic data during training by Huber et al.^24^. They benchmarked different DH-NN against feature-based methods for laparoscopic camera motion estimation, aiming at camera automation, independent from object and instrument motion. In another application, DH-NN-based mosaicking was used to enable the expansion of the field-of-view in fetoscopic image data^25^. Moving objects and depth differences are challenging for DH-NN as the whole image contributes to homography estimation. Therefore, an outlier rejection, known from RANSAC, was introduced for content-aware unsupervised deep homography estimation, that was trained and evaluated with non-clinical data^26^. Nie et al.^27^ extended the depth-awareness of DH-NN with the estimation of a multi-grid deformation mesh for handling real-world images with parallax (MG-DHNN).

### Contribution

The main contributions of this work are threefold. Firstly, a non-rigid DH-NN and feature-detector-descriptor algorithms for single and multi-homography estimation are evaluated for the alignment of laparoscopic images.

Secondly, a free available dataset of manually annotated landmarks over 750 frames of a laparoscopic scene with occlusions and large tissue deformations is provided. Other ground truth datasets for non-rigid image registration mentioned in the literature are not published or not available anymore.

Finally, to the best of our knowledge, registration of laparoscopic video and static HSI data for augmentation with physiological tissue information is a novel approach.

## Methods

This section describes the laparoscopic videos used for evaluation and training, as well as the formation of the ground truth data. The human dataset used in our study is publicly available and all methods were carried out in accordance with relevant guidelines and regulations. All implemented steps from image preprocessing, keypoint detection, description, and matching until image transformation are defined. Finally, the quantitative evaluation with ground truth data and clinical measurements for qualitative assessment are presented.

The feature-detector-descriptor algorithms were implemented on a Windows machine (2.11 GHz Intel Core i7) with Python 3.6 using OpenCV^28^ and executed on the CPU, whereas the deep homography methods were performed on a GPU (NVIDIA MX150) using TensorFlow 1.13.

### Ground truth dataset

Based on a laparoscopic video, recorded during hybrid esophagectomy, the ground truth dataset was built. Details about the case and procedure are reported in a video publication^29^. The raw video lasts 106 min at 25 frames per second with an image size of 854 × 480 pixels. Ten scenes with 750 frames (30 s) each, were randomly extracted from the raw video. One out of the ten scenes was selected for manual annotation because it included surgical instruments, organ motion due to breathing and surgical manipulations, as well as perspective changes.

For the manual annotation of corresponding points between frames, the open-source *Computer Vision Annotation Tool* (CVAT) was used. It supports the import and export of the annotations as XML-file for further processing in other environments. The video data and ground truth annotations are made publicly available in Supplementary Data S1.

**Figure 1:**
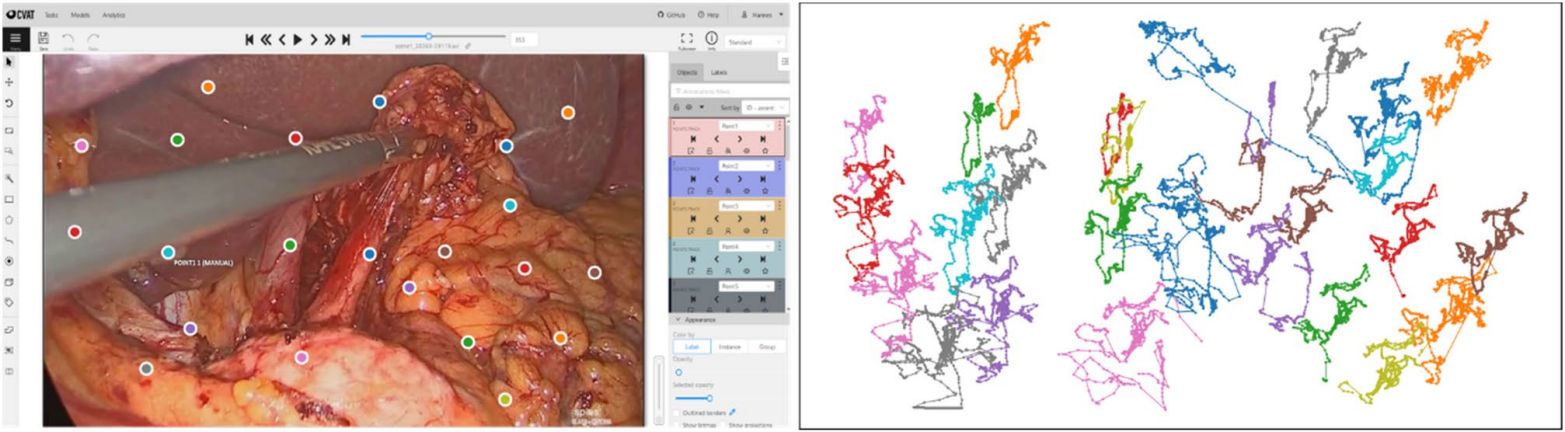
Left: Graphical user interface of CVAT showing a frame with landmark occlusion due to a laparoscopic instrument. Right: Motion paths of the 28 manually annotated landmarks used as ground truth during all 750 frames of the scene.

Twenty-eight evenly spaced landmarks were selected. All landmarks drawn on each frame are shown in Supplementary Video S2. Due to the occlusion of individual landmarks in single frames, the average of visible points per frame is 20. The motion paths of all 28 landmarks during the entire scene are shown in Fig. 1.

### Image preprocessing of video frames

Image preprocessing is used to increase the number of salient features and to reduce the impact of noise, glare artifacts, and illumination changes. First, the green channel of the color image was selected as proposed by Sieler et al.^15^ and confirmed in preliminary tests. This reduced registration error by 36% for ORB1000 and 4% for the other methods tested.

Glare artifacts and dark areas were masked by setting the 20th and 80th percentile of the maximum value range to 0, followed by a morphological erosion of the remaining image area with an elliptical kernel of 5 × 5 pixels. This mask is hereafter referred to as glare mask and reduced registration error by up to 35%.

For image smoothing, the single-channel image was convolved with a normalized 5 × 5 box filter. Afterward, Contrast Limited Adaptive Histogram Equalization (CLAHE) with a tile size of 8 × 8 and a clip limit of 2.0 was performed^30^. Preliminary tests showed, that CLAHE reduced registration error by 13–19% for all tested methods.

### Keypoint detection, description, and matching

Keypoint detection and description were performed on the preprocessed image within the glare mask. Three different methods were investigated: Oriented FAST and Rotated BRIEF with a maximum number of 1000 keypoints (ORB1000)^31^, Accelerated-KAZE (A-KAZE)^32^, and Binary Robust Invariant Scalable Keypoints (BRISK)^33^. Additionally, all methods were tested in combination with Boosted Efficient Binary Local Image Descriptor (BEBLID) with 256 and 512 bits^17^.

For feature matching, the Hamming distance with crosscheck was applied and 20% of the best matches were used for further processing. This resulted in 200 features per frame with ORB1000. The maximum number of features per image is not fixed for A-KAZE and BRISK. An average of about 400 and 500 best matches were used for A-KAZE and BRISK respectively.

### Outlier rejection and image transformation

Image transformation was performed with: (a) perspective transformation using a single homography (SH) from RANSAC^34^ with a reprojection error threshold of 5, (b) hierarchical multi-affine (HMA) approach^18^, (c) image deformation with affine Moving Least Squares (MLS)^12^, and (d) multi-grid deep homography estimation (MG-DHNN)^27^.

The latter three were tested to compensate for local image deformations due to breathing and manipulations from surgical instruments. HMA uses geometrical constraints to create multiple regions with similar keypoints, that are further divided and/or expanded if appropriate. Each image area covered by such a region has an individual homography estimated with RANSAC. The developed Python implementation is based on MATLAB scripts from https://github.com/gustavhafen/HMA and the implemented combination of SH and HMA is described in section Quality Check and Postprocessing. Dense image deformation can be obtained with MLS. Therefore, the matched keypoints are used to obtain an affine transformation for the vertices of a uniform grid and the pixels between are bilinearly interpolated. As a trade-off between deformation accuracy and computation time, a grid density of 20% was chosen, as suggested in Schaefer et al.^12^.

To overcome the limitations of feature-based methods in scenes with low texture or unevenly distributed features, the MG-DHNN (based on https://github.com/nie-lang/Multi-Grid-Deep-Homogarphy) was tested for elastic image registration. The MG-DHNN was chosen because it was trained on images with parallax from UDIS-D^35^ and provides a deformation mesh, suitable to compensate local tissue displacement. Since it can be trained fully unsupervised, we used real-world laparoscopic images for fine-tuning with additional 10 k iterations and a batch size of 4. No depth information was used and therefore the weights for content and shape were set to 1 and 0, respectively. In Nie et al.^27^, the training parameters are described in detail. The training set contains 6034 image pairs (a and b) extracted from the video described in section A (excluding the annotated scene used for the evaluation) and one video from the LapChole dataset^36^. Every 30th frame is selected as image a, while image b is sequentially selected at 50, 100, 150, 200, 250, or 300 frames distance. Image pairs without or very low resemblance were manually discarded. No image preprocessing was performed for the use of the MG-DHNN.

### Quantitative evaluation with ground truth

**Figure 2:**
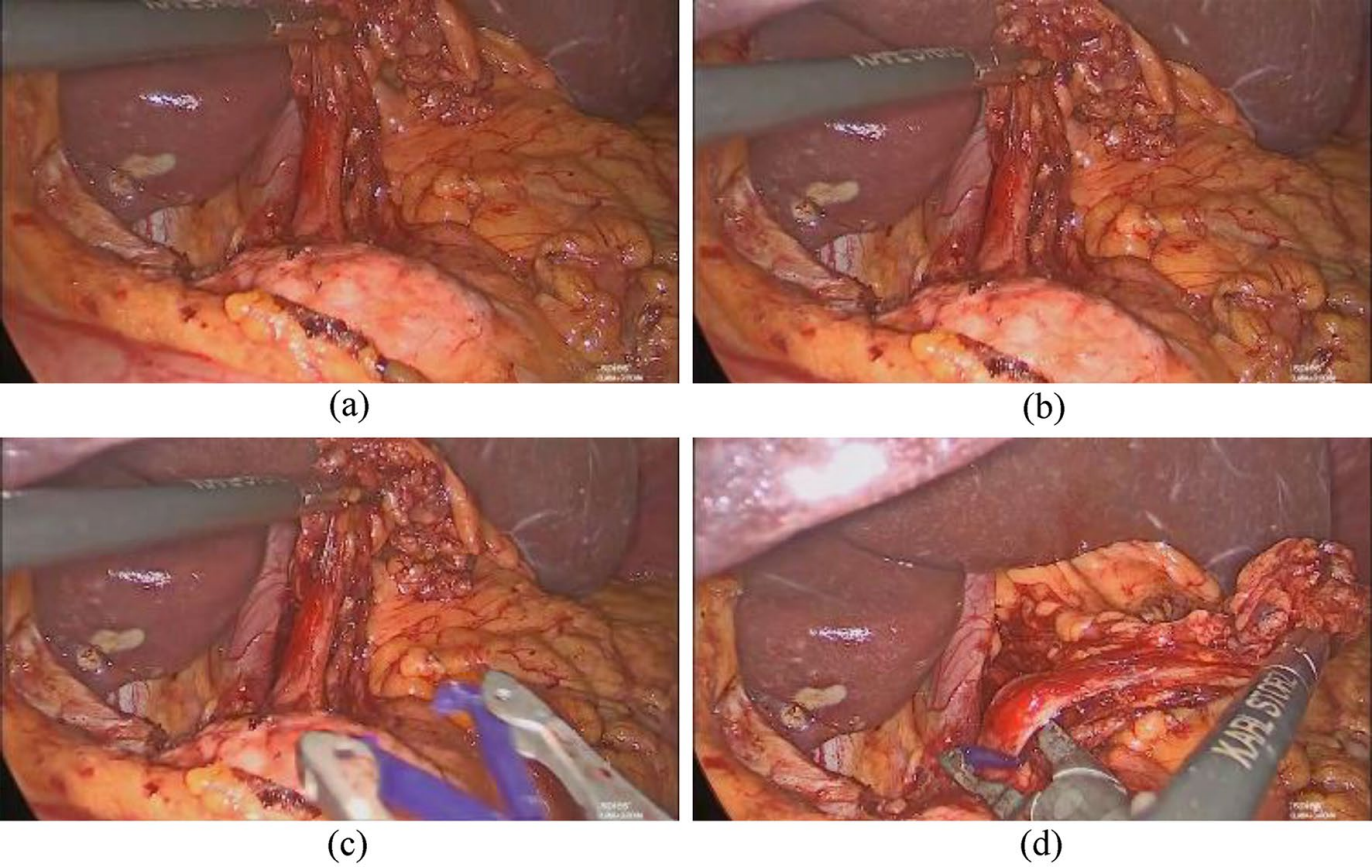
Images used as start frames for the four scenes. Frame number (**a**) 20, (**b**) 200, (**c**) 400, and (**d**) 600 of the video.

The estimated transformations were quantitatively evaluated with the manual ground truth annotations. To obtain a more general validation, four frames (number 20, 200, 400, and 600) were selected as start frames, shown in Fig. 2. Image registration was calculated between a start frame and each subsequent frame until frame 750, referred to as a scene.

The estimated transformation was applied to the ground truth points of the start frame and the reprojection errors (RE) in pixel were calculated. Each RE was normalized by the individual moved distance. Afterward, the RE and normalized RE were averaged over the current frame, scene, and all four scenes. Thus, the normalized RE of a point after movement and without any image transformation (identity homography) is 1.

The Structural Similarity Index Measure (SSIM)^37^ was calculated between the start frame and the transformed frames.

### Quality check and postprocessing for visualization

Several quality checks were implemented to avoid erroneous visualizations during practical use. It is pointed out, that these steps were not applied during the quantitative evaluation of RE and SSIM. Further image processing was already stopped after feature matching if the average matching distance was higher than 35, 55, and 65 for ORB1000, A-KAZE, and BRISK, respectively. In those cases, the original current frame was shown. These thresholds were adjusted for each method individually and correspond to a resulting RANSAC inlier ratio of 0.6, considering a RANSAC inlier is defined by a reprojection error of less than 5.

The obtained HMA regions are not covering the full image, for example, due to missing or poor features in some areas. Therefore, the start frame was transformed with the homography of each valid HMA region and the homography for the full image separately. An HMA region was valid if (a) the area was greater than 2% of the image area and (b) the sum of absolute differences between the local homography and the SH was less than 50. Difference images of the start frame transformations and the current frame were calculated and a morphological closing with a 35 × 35 elliptical kernel was performed. Based on these difference images, the transformation with the lowest error was selected for each pixel. This procedure can be easily extended with other methods, for example, image deformations obtained from MLS or MG-DHNN. The processing pipeline is illustrated as flow chart in Supplementary Fig. S3.

The lowest error for each pixel is used to define the combined error image. This was used to create a binary mask where the error was higher than 10% of the maximum value range. Finally, a median filter (35 pixels) was applied to this mask that indicates for each pixel whether the result of the transformations or the original current frame was shown.

### Qualitative evaluation: intraoperative laparoscopic hyperspectral imaging

In-vivo hyperspectral imaging (HSI) and video data were acquired in one patient with the medical approved successor of the laparoscopic system introduced in Köhler et al.^5^. Video data with an image size of 1920 × 1080 pixels at 30 frames per second and hyperspectral data with 720 × 540 pixels in spatial dimension were recorded from two image sensors simultaneously. The latter consists of 100 spectral channels in the wavelength range from 500 to 1000 nm, resulting in a spectral resolution of 5 nm. As mentioned in the introduction, this spectral information was used to quantify the tissue oxygenation and visualize the same as a static false-color image for the intraoperative assessment of organ perfusion. The measurements have obtained ethics approval by the Ethics Committee of the University Leipzig under 393/16-ek and were conducted according to the Declaration of Helsinki.

**Figure 3:**
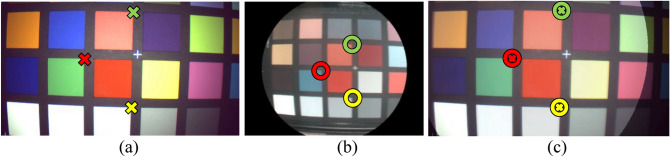
One-time registration of color video and hyperspectral data. (**a**) Image from the color sensor with three exemplary markers. (**b**) Pseudocolor image reconstructed during HSI of the same object. Missing information in the blue spectral range causes diverging coloration. Corresponding points to the markers in (**a**) are labeled with circles. (**c**) Semitransparent overlay of both images after perspective transformation based on 25 manually annotated points.

Two main steps are required for the augmentation of the video with the oxygenation maps obtained from HSI. First, a one-time registration based on manual annotation of both modalities has to be performed to align the video and hyperspectral images. The result of this first step is illustrated in Fig. 3 and a detailed description can be found in Köhler et al.^5^. This calibration does not have to be repeated before surgery because the arrangement of the color and hyperspectral image sensor is fixed inside the camera housing. Second, during the HSI record, one image of the video is saved as a start frame and will be used for the registration with all consecutive frames. The video is augmented by applying the transformations found in steps one and two to the oxygenation map and creating a semitransparent overlay with the grayscale image of the current video frame. Due to regulatory restrictions, the augmentation process was performed postoperatively.

### Ethical approval

We confirm that all methods were performed in accordance with the relevant guidelines and regulations.

## Results

### Quantitative results

A mean movement of 41.8 pixels was obtained for the annotated visible points, averaged over all frames of each scene, which is equivalent to the mean RE without any transformation (identity homography). The mean RE and mean normalized RE of the perspective transformation estimated with RANSAC from the ground truth annotations were 8.44 and 0.27, respectively.

A-KAZE, ORB1000, and BRISK showed similar mean normalized RE (< 0.38) for the estimation of an SH and the HMA method. Although the mean normalized RE inside the HMA clusters were reduced by 47% and 39% compared to the single homography for A-KAZE and BRISK respectively, the improvement for the entire image was marginal.

All three feature-detector-descriptor algorithms gave higher errors for the MLS method, only A-KAZE and BRISK yielded a mean normalized RE below 1. Keypoint description with BEBLID 512 reduced the mean normalized RE for ORB1000 and BRISK in combination with MLS by 30% and 34% respectively.

The MG-DHNN methods resulted in higher mean RE compared to the algorithms based on SH but performed better than the MLS methods. Fine-tuning of the MG-DHNN reduced the mean RE by 35%, however, SSIM was not improved.

Mean SSIM was similar for all methods, with the lowest value for the identity homography (0.431) and the highest for the SH calculated with BRISK (0.529), which even outperformed the SH based on manual annotation (0.513). The means of RE, normalized RE, and SSIM for all tested methods are given in Table 1.

**Table 1:** Quantitative comparison of the tested methods.

Method	Mean RE (↓)			Mean normalized RE (↓)			Mean SSIM (↑)		
No transformation	41.77			1			0.431		
*Manual annotation*									
SH	8.44			0.267			0.513		
MLS	6.20			0.257			0.531		
BEBLID	w/o	256	512	w/o	256	512	w/o	256	512
*A-KAZE*									
SH	10.87	**10.77**	10.88	**0.346**	0.350	0.355	0.526	0.528	*0.528*
MLS	25.13	44.89	34.92	0.690	1.104	0.851	0.519	0.489	0.504
SH + HMA	10.88	*10.82*	10.89	*0.346*	0.355	0.357	0.518	0.521	0.521
*ORB 1000*									
SH	11.54	12.20	12.16	0.374	0.395	0.390	0.520	0.523	0.523
MLS	57.17	46.01	38.98	1.465	1.214	1.023	0.480	0.494	0.503
SH + HMA	11.54	12.21	12.26	0.374	0.395	0.391	0.517	0.520	0.519
*BRISK*									
SH	11.35	11.52	11.51	0.366	0.367	0.361	**0.529**	0.527	0.527
MLS	51.02	38.41	30.98	1.240	1.006	0.820	0.487	0.494	0.511
SH + HMA	11.36	12.68	11.52	0.365	0.380	0.361	0.521	0.520	0.520
MG-DHNN^25^	20.18			0.549			0.515		
MG-DHNN fine-tuned	13.17			0.408			0.514		

First and second-best solution are printed in bold and italic respectively.

**Figure 4:**
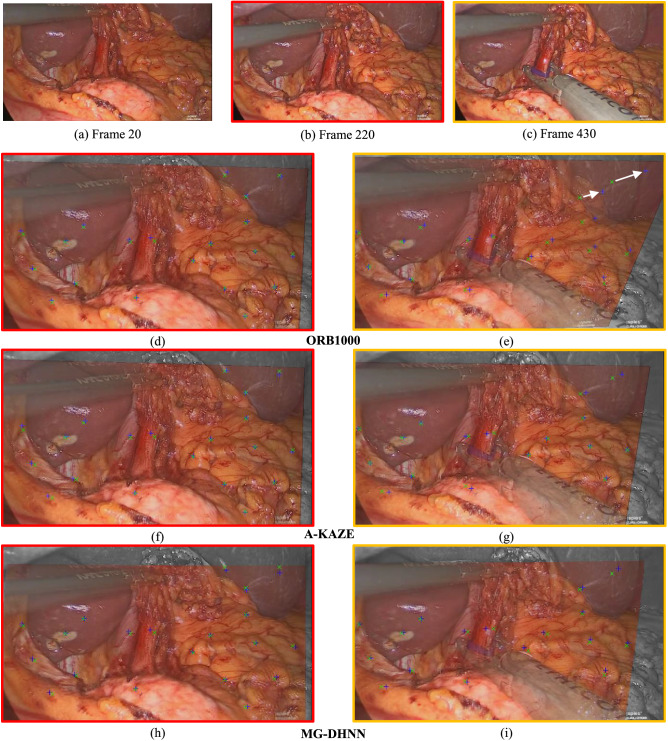
Image registration results with the single homography method. (**a**) Frame 20 is used as the start frame of scene 1. (**b**) Frame 220 with small perspective changes, instrument movement, and organ deformation in relation to frame 20. (**c**) Frame 430 shows occlusions from an instrument and organ deformation due to manipulation compared with frame 20. (**d**)–(**i**) Semitransparent overlay of the transformed start frame 20 and the current frames 220 (left column, red) and 430 (right column, yellow) for ORB1000 (**d**, **e**), A-KAZE (**f**, **g**), and MG-DHNN (**h**, **i**). Green crosses indicate the ground truth and blue crosses the estimated position of the annotated points. White arrows highlight large registration errors and non-overlapping regions are shown in greyscale. The normalized RE averaged over the annotations of the shown frame are 0.1 for **d**, **f**, and **g**; 0.26 for **e**; 0.13 for **h** and **i**.

The registration results of start frame 20 with two exemplary frames, 220 and 430, obtained from SH (ORB1000 and A-KAZE) and the original MG-DHNN are shown in Fig. 4. Displacements between ground truth and estimated transformation are indicated with crosses. Despite a larger RE in frame 430 with ORB1000, all three methods gave similar results for both frames, even under tissue deformation and partial occlusion.

**Figure 5:**
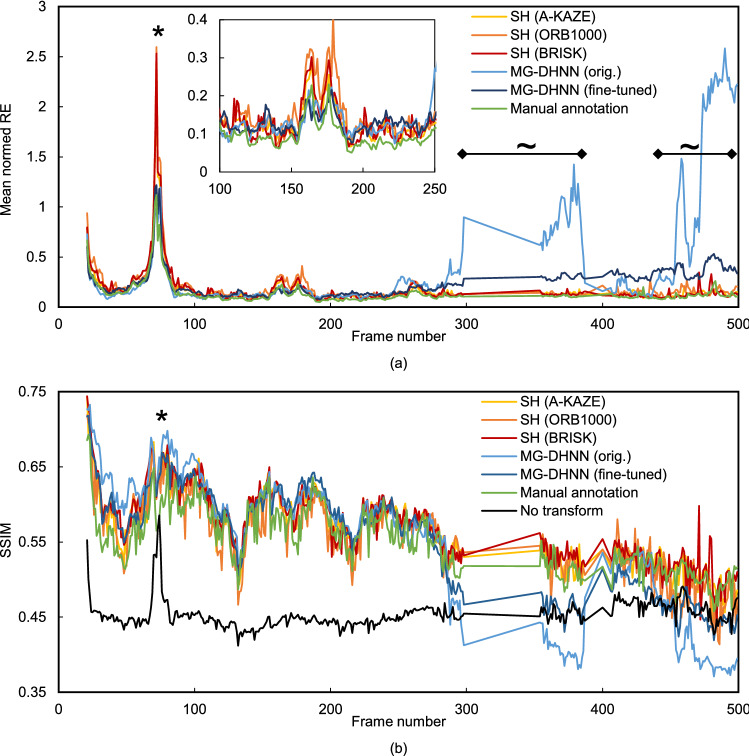
(**a**) Mean normalized RE and (**b**) SSIM of start frame 20 and all consecutive frames with more than 15 corresponding manual annotations. Mean normalized RE and SSIM are shown for single homography estimation (A-KAZE, ORB1000, BRISK, and manual annotation) and MG-DHNN (original and fine-tuned). Additionally, SSIM is shown without any applied transformation. Frames labeled with * are very similar to the start frame and show a high SSIM without transformation. The mean normalized RE shows higher values for these frames because of the very small moved distances of the manual annotations. However, the SSIM of the transformed frames is still higher than without transformation. Frames labeled with ~ include tissue deformations caused by surgical instruments. From frame number 390 on, large parts of the image are partially occluded by two instruments in the field of view.

For the comparison of the tested SH and MG-DHNN methods and the ground truth over consecutive frames, the mean normalized RE and SSIM of the first scene are plotted in Fig. 5. Additionally, the registration of start frame 20 with the subsequent frames based on SH and A-KAZE is available in Supplementary Video S4. A more detailed representation of the RE and the movement of each annotation is given in Supplementary Fig. S5 for ORB1000 and SH. In frames with small tissue deformation, the original MG-DHNN shows similar or better results compared with the SH methods. However, if the tissue deformation is stronger, the mean normalized RE is greater than 1 and SSIM is even lower than without transformation. This was not obtained for the SH methods, but the MLS with ORB1000 and BRISK. The fine-tuned MG-DHNN performed much better than the original MG-DHNN in frames with strong tissue deformation, but SH methods were more robust.

**Figure 6:**
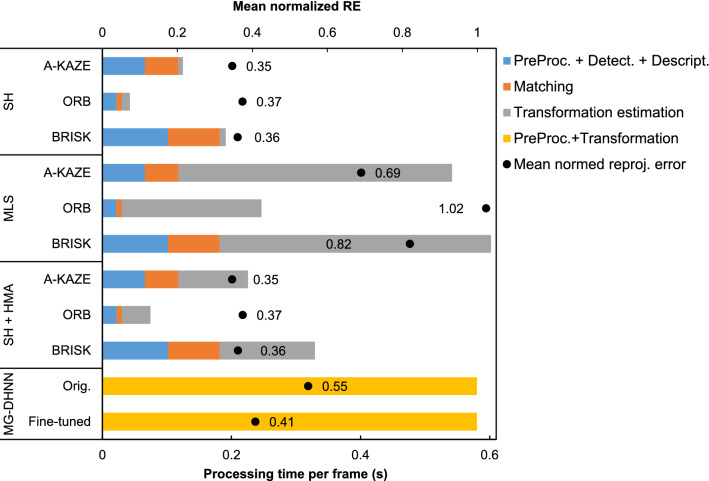
Mean processing time per frame and mean normalized reprojection error (RE) for each transformation and feature-detector-descriptor method. ORB is the short form of ORB1000.

ORB1000 was the fastest feature-detector-descriptor with 22 ms (± 0.3 ms) for preprocessing, detection, and description, followed by A-KAZE and BRISK with 66 ms (± 2 ms) and 102 ms (± 2.6 ms) respectively. Feature matching was performed in 8 ms (± 0.1 ms) for ORB1000, 52 ms (± 5 ms) for A-KAZE, and 80 ms (± 7.9 ms) for BRISK features. Estimating a single homography was faster than the HMA or MLS method for all three feature-detector-descriptor algorithms. Processing a frame faster than 50 ms (20 fps) was only possible for the estimation of a single homography with ORB1000 (43 ms ± 5.8 ms). Transforming an image with the MG-DHNN was performed in 580 ms (± 11.4 ms). The mean normalized RE and mean processing times are shown in Fig. 6 for each tested transformation method using the respective BEBLID setting resulting in the lowest normalized RE.

### Qualitative results with HSI data

**Figure 7:**
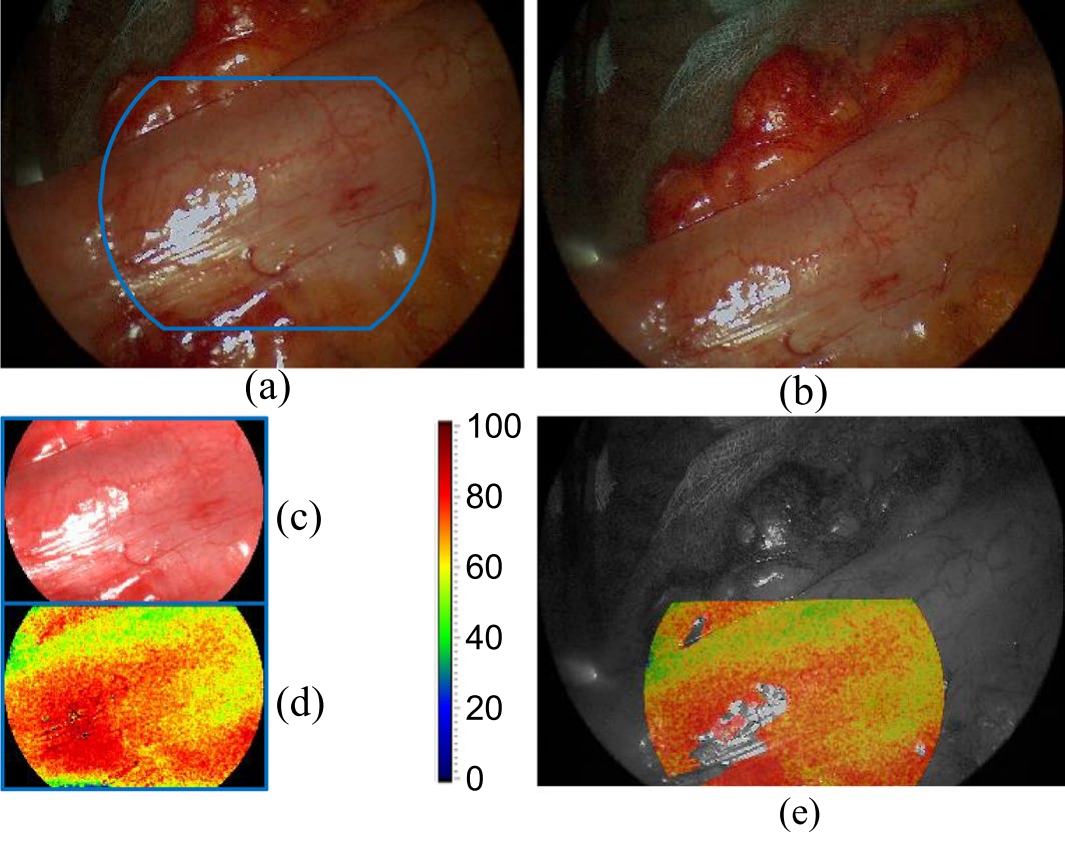
Augmentation of in-vivo video data with HSI. (**a**) Start frame from the color sensor acquired during the HSI record. The blue outline indicates the region visible in HSI. (**b**) An exemplary target frame of the video, 20 s after HSI was performed. (**c**) Pseudocolor image reconstructed from hyperspectral data. (**d**) Static color map representing the tissue oxygenation according to the color scale. (**e**) Semitransparent overlay of the oxygenation map and the grayscale of the target frame after registration and postprocessing.

The developed preprocessing and registration process enabled the augmentation of in-vivo laparoscopic video data with tissue oxygenation maps from HSI. Figure 7 shows the acquired image data and the resulting overlay of both modalities after perspective transformation with an SH found with ORB1000 based on the start and target frame of the video. Regions with an intensity difference higher than the error threshold were excluded from the overlay during postprocessing. This prevents misleading visualization in cases with strong glare artifacts (Fig. 7e) or surgical instruments in the field of view. Although the preprocessing was optimized on the ground truth dataset of another organ recorded with a standard laparoscope, robust and accurate augmentation of the multimodal image data was observed. There were no strong tissue deformations or surgical instruments in the available scene. Augmentation with other physiological tissue maps like hemoglobin or water content can be selected by the user during runtime because they are calculated from the same hyperspectral data, a new record is not needed. Image registration is only performed between the start frame and the current frame, which makes the method robust against temporary occlusion or changes in the field of view. In contrast, strong illumination changes or organ deformations are more challenging for this approach in comparison to feature tracking methods. Push-broom HSI takes a few seconds, thus motion artifacts can occur during data acquisition and affect the accuracy of the overlay.

## Discussion

Minimally-invasive procedures are becoming more common in recent years. Haptic perception is missing during MIS, therefore imaging technology has a key role in the improvement of patient safety. The intraoperative augmentation of different modalities, such as color and perfusion imaging, can provide additional support. Augmenting video data and static images requires real-time registration, which is especially challenging in laparoscopy due to tissue deformations, occlusion, or non-planar surfaces.

In this work, a ground truth dataset of 750 frames with an average of 20 manually annotated points per frame is made publicly available. Thus, the dataset can be easily extended and modified by others. It can be used for the validation of non-rigid image registration methods under challenging real-world conditions. Although the proposed dataset consists of only one scene, it includes much more annotated frames than previous validation data used by others^8,18^. DH-NN typically require thousands of images for training. Thus synthetic data for supervised training or similarity measures for unsupervised training are needed. However, for the evaluation of registration accuracy, RE from manual annotations are more expressive than similarity metrics.

Image preprocessing improves the performance of feature-detector-descriptor methods on laparoscopic data substantially. The green channel gave better results in preliminary tests compared to the typically used grayscale, which is in line with the findings by Sieler et al.^15^. Additionally, these tests showed that CLAHE reduces the effect of illumination changes, resulting in lower RE for all tested feature-based methods. A-KAZE outperformed ORB1000, BRISK, and MG-DHNN in terms of RE and normalized RE for the single and multi-homography approaches. Image registration at video rate (23 frames per second) was achieved with the single homography estimation based on ORB1000.

Although the MLS method outperformed the single homography for the manual annotation (see Table 1), it resulted in the highest RE and lowest SSIM of all tested methods. This might be due to the non-uniform distribution of detected features and missing outlier elimination, such as RANSAC, which leads to high RE in frames with strong tissue deformation. The MLS method was successfully used by Selka et al.^8^ with SURF for the detection of more uniform distributed features. However, the validation dataset differs from the one presented here and a comparison with single homography methods was not performed. The processing times needed for MLS-based transformation estimation with 200 features reported in Selka et al.^8^ and this work are almost equal (0.18 s and 0.2 s).

In accordance with Puerto-Souza et al.^18^, the registration of clusters found by HMA in combination with A-KAZE and BRISK was improved compared to the SH result of the same region. Using the homographies obtained by HMA for image alignment beyond the clusters did not result in substantial improvement. Alternative feature-detectors, such as SIFT and SURF, might improve the results of MLS and HMA due to a higher number of evenly distributed features, but require more time for total image matching^13^.

The original MG-DHNN trained on non-medical images showed similar or better results than the single homography methods for frames with little deformation (see Figs. 4, 5). High errors occurred in frames with larger deformations in parts of the image. Fine-tuning with laparoscopic data improved RE and SSIM substantially for these frames, but did not reach the performance of the single homography. This is contrary to the findings in Huber et al.^24^, where DH-NN outperformed the feature-based methods SIFT, SURF and ORB. One reason might be the missing preprocessing, which is a disadvantage for the latter. Furthermore, the number of frames with large deformations included in the validation dataset, the error metric, and the aim to estimate the camera movement, could explain the different results. In this work, MG-DHNN was the slowest of the tested methods, but processing of two frames is possible in 96 ms on a GPU with NVIDIA RTX 2080 Ti^27^. Less complex DH-NN can be faster than feature-based methods even on a CPU^24^. As the processing time is no disadvantage of DH-NN necessarily, future training data should improve the robustness of these methods in images with large local deformations.

Depending on the image depth, 12 to 17 pixels of a frame represent 1 mm. Besides MLS and the original MG-DHNN, all tested methods provide a mean RE lower than 1 mm.

## Conclusion

Three feature-detector-descriptor algorithms were tested in combination with single and multi-homography methods and compared with a multi-grid DH-NN on manually annotated laparoscopic images. Video-rate image registration with submillimeter accuracy was possible using SH transformation based on ORB1000. Future work should include multiple ground truth data from different imaging systems and procedures to evaluate the transferability of the results found. DH-NN methods are promising for non-rigid image registration, provided that suitable training data will be available. Hybrid approaches, combining the advantages of feature-based outlier rejection and MG-DHNN could overcome the current limitations for routine clinical use.

## Supplementary Information

Supplementary Information 1.

Supplementary Video 1.

Supplementary Information 2.

Supplementary Video 2.

Supplementary Information 3.

Supplementary Information 4.

## Data Availability

All data generated or analysed during this study are included in this published article and its Supplementary Information files.
