# Supplementary material for: Comparison of image registration methods for combining laparoscopic video and spectral image data
Source: Sci Rep. 2022 Sep 30;12:16459. doi: 10.1038/s41598-022-20816-1 (PMC9525266; doi:10.1038/s41598-022-20816-1)

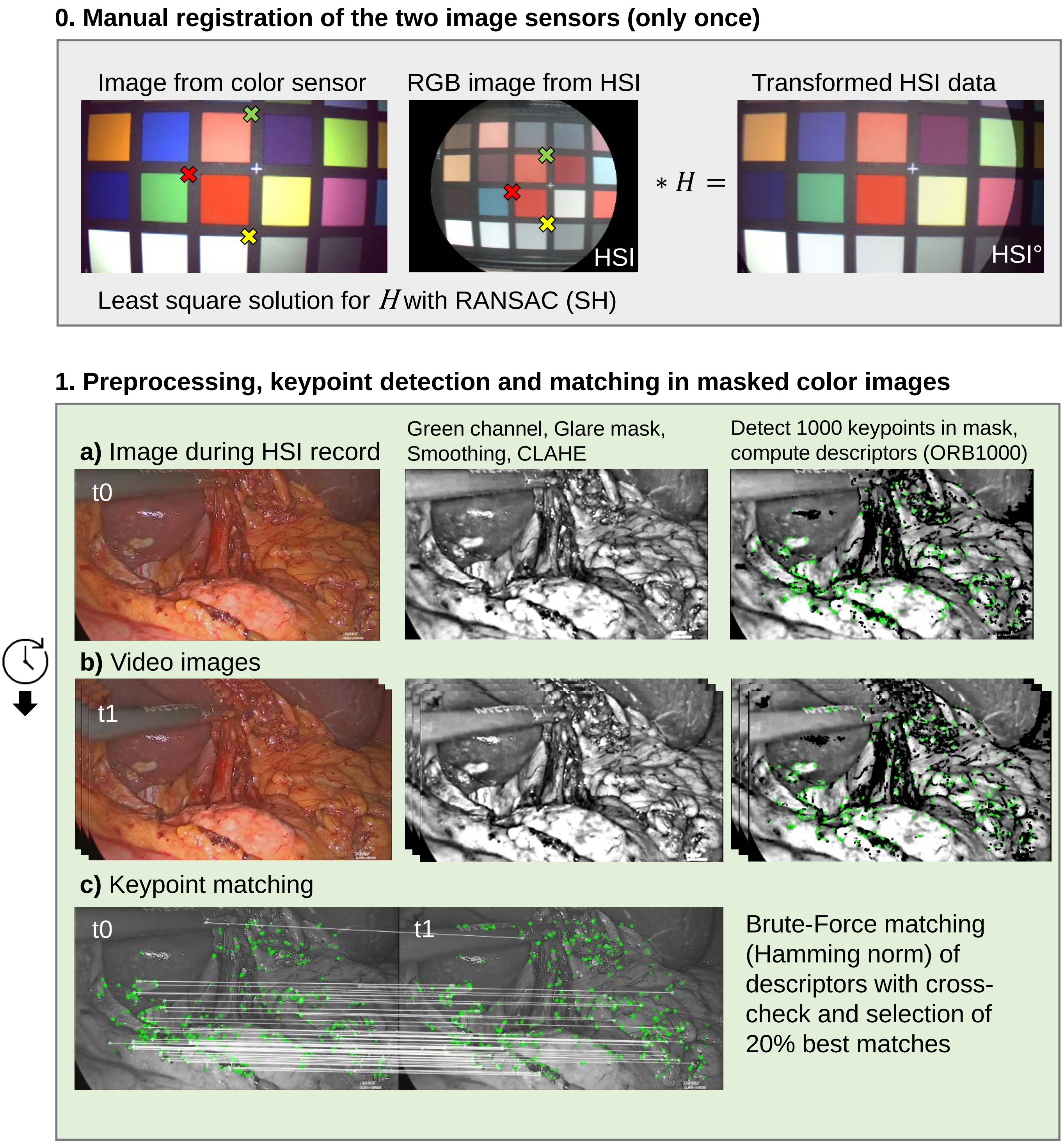
0. Manual registration of the two image sensors (only once)
Image from color sensor
RGB image from HSI
HSI
* H =
Transformed HSI data
HSI°
Least square solution for H with RANSAC (SH)
1. Preprocessing, keypoint detection and matching in masked color images
a) Image during HSI record
t0
Green channel, Glare mask, Smoothing, CLAHE
Detect 1000 keypoints in mask, compute descriptors (ORB1000)
b) Video images
t1
c) Keypoint matching
t0
t1
Brute-Force matching (Hamming norm) of descriptors with cross-check and selection of 20% best matches

2. Calculation of transformation matrices from point pairs

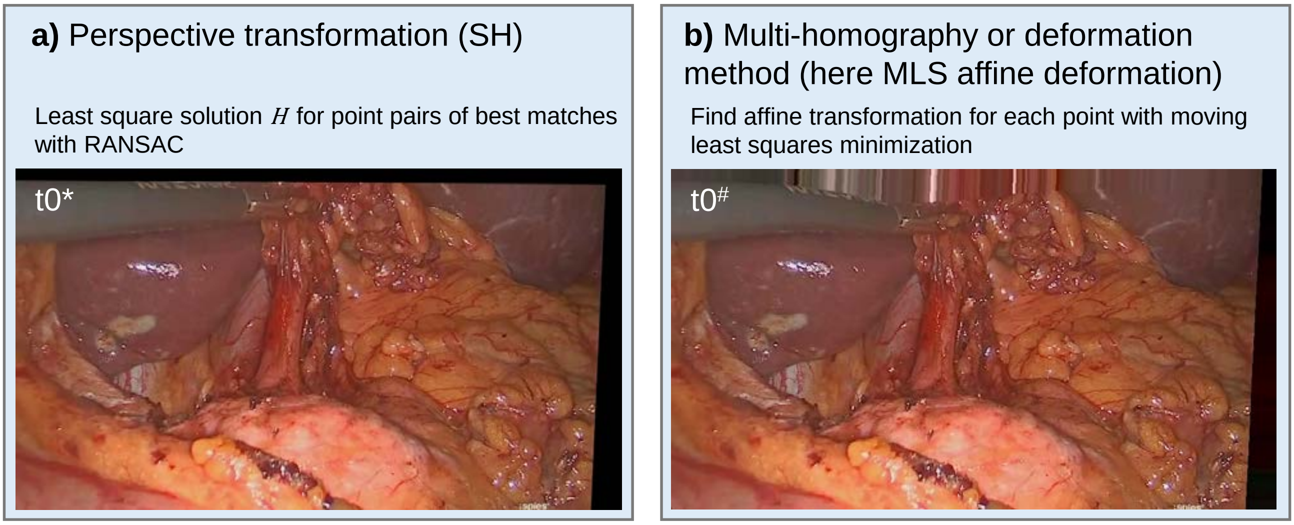

3. Overlay of HSI data on video

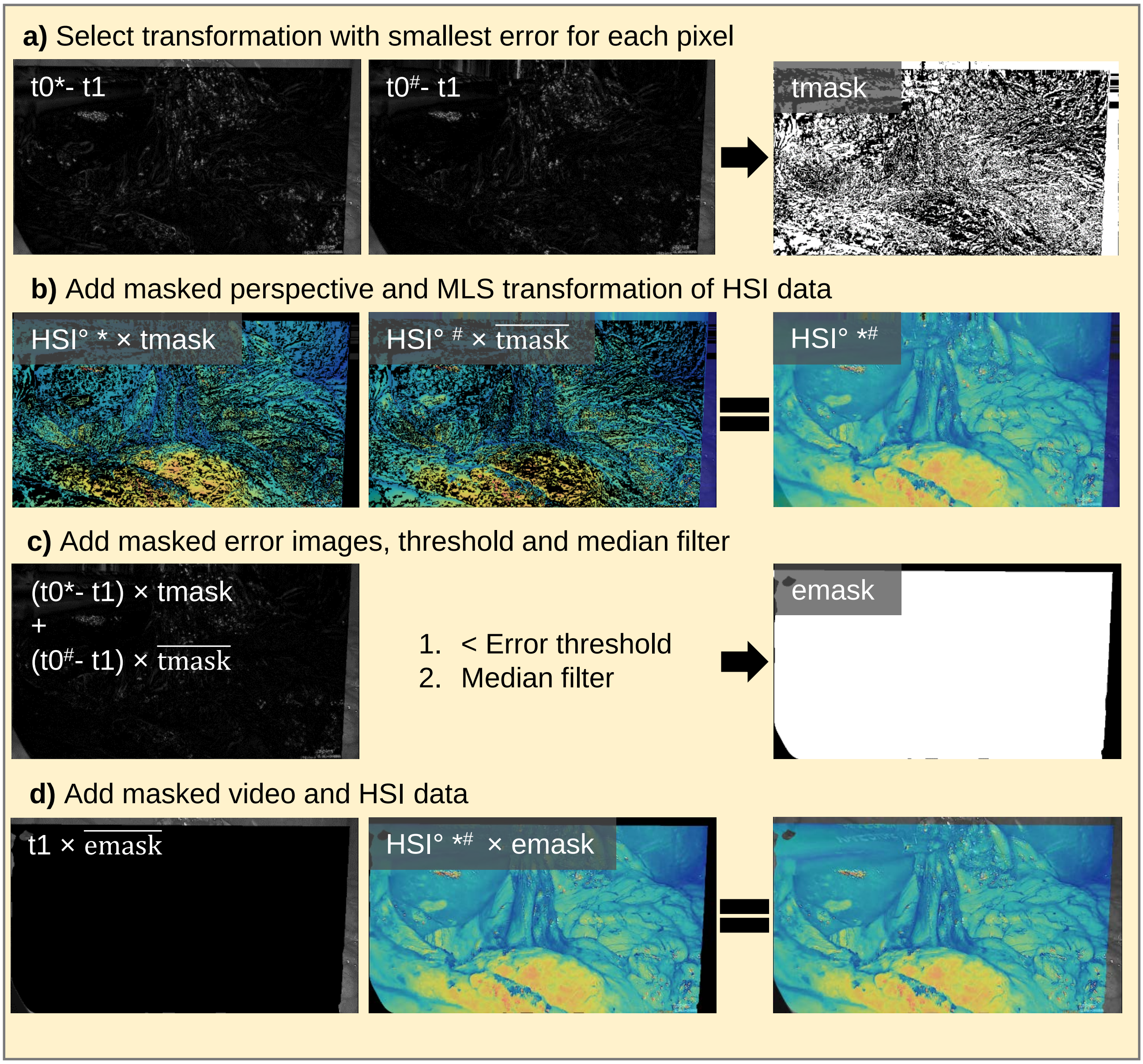

Supplement: Supplementary file 3 — Supplementary Information 2. [file 41598_2022_20816_MOESM3_ESM.pdf]
