# Supplementary figures and images for: Comparison of image registration methods for combining laparoscopic video and spectral image data
Source: Sci Rep. 2022 Sep 30;12:16459. doi: 10.1038/s41598-022-20816-1 (PMC9525266; doi:10.1038/s41598-022-20816-1)

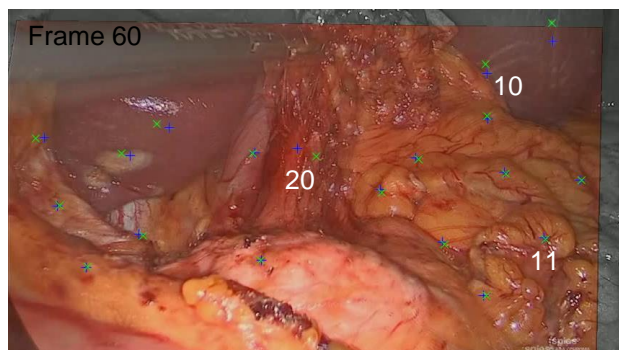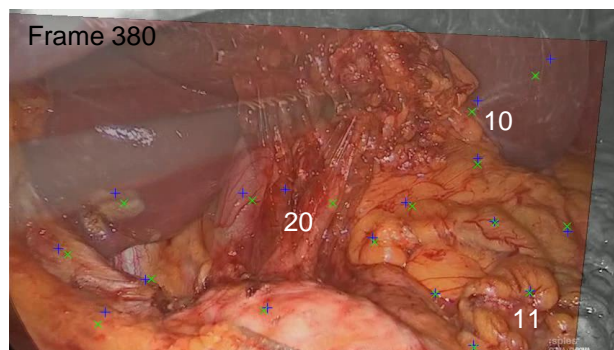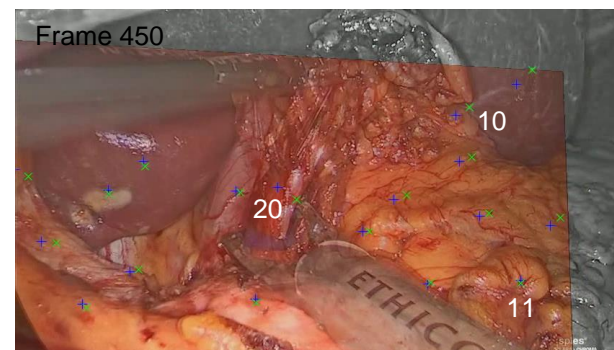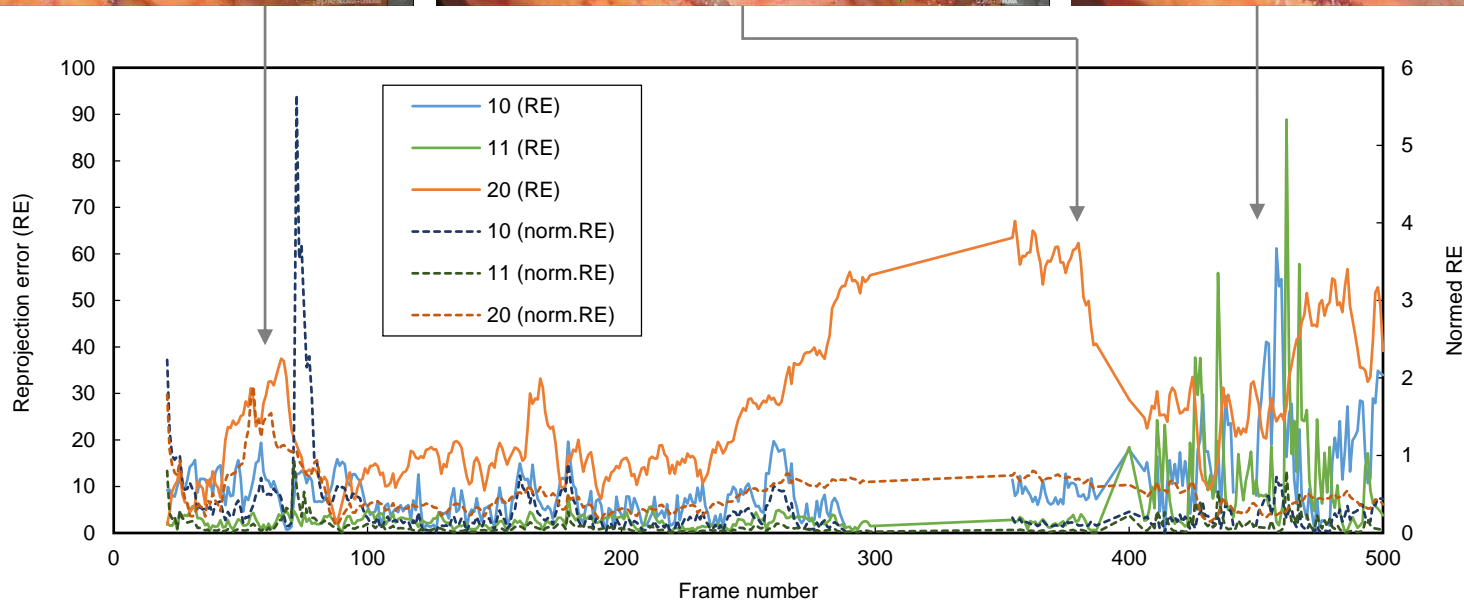

|           |      | Marker ID | 7     | 9     | 10          | 11          | 12    | 13    | 14    | 15    | 16    | 17    | 18    | 19    | 20           | 21    | 22    | 23    | 24    | 25    | 26    | 27    |
|-----------|------|-----------|-------|-------|-------------|-------------|-------|-------|-------|-------|-------|-------|-------|-------|--------------|-------|-------|-------|-------|-------|-------|-------|
| RE        | Mean |           | 11.04 | 5.75  | <b>9.87</b> | <b>4.85</b> | 6.10  | 3.20  | 5.73  | 4.55  | 8.36  | 7.46  | 2.55  | 5.43  | <b>26.30</b> | 16.71 | 3.44  | 9.47  | 4.21  | 6.75  | 6.26  | 7.60  |
|           | SD   |           | 6.12  | 4.29  | 7.97        | 7.89        | 2.85  | 4.28  | 3.14  | 3.78  | 11.05 | 5.07  | 1.81  | 4.37  | 15.61        | 13.53 | 4.02  | 5.13  | 3.32  | 6.82  | 4.34  | 4.89  |
| Distance  | Mean |           | 36.38 | 46.77 | 47.30       | 62.42       | 46.22 | 54.60 | 46.46 | 49.47 | 55.21 | 52.39 | 48.80 | 51.59 | 55.04        | 49.53 | 52.35 | 39.13 | 51.79 | 54.63 | 44.54 | 42.88 |
|           | SD   |           | 14.35 | 22.97 | 22.77       | 28.56       | 21.06 | 22.81 | 20.47 | 20.02 | 30.62 | 24.13 | 21.24 | 22.42 | 25.81        | 24.79 | 23.48 | 19.67 | 22.19 | 23.75 | 21.46 | 21.89 |
| Normed RE | Mean |           | 0.50  | 0.16  | <b>0.30</b> | <b>0.08</b> | 0.17  | 0.07  | 0.16  | 0.10  | 0.16  | 0.16  | 0.07  | 0.14  | <b>0.51</b>  | 0.52  | 0.08  | 0.32  | 0.09  | 0.13  | 0.20  | 0.26  |
|           | SD   |           | 0.81  | 0.19  | 0.47        | 0.11        | 0.24  | 0.08  | 0.18  | 0.09  | 0.19  | 0.11  | 0.11  | 0.17  | 0.27         | 1.20  | 0.08  | 0.32  | 0.07  | 0.11  | 0.40  | 0.37  |

Supplement: Supplementary file 5 — Supplementary Information 3. [file 41598_2022_20816_MOESM5_ESM.pdf]
