# Supplementary material for: Comparison of image registration methods for combining laparoscopic video and spectral image data
Source: Sci Rep. 2022 Sep 30;12:16459. doi: 10.1038/s41598-022-20816-1 (PMC9525266; doi:10.1038/s41598-022-20816-1)
Supplement: Supplementary file 6 — Supplementary Information 4. [file 41598_2022_20816_MOESM6_ESM.docx]

**Supplementary Information**

Comparison of Image Registration Methods for Combining Laparoscopic Video and Spectral Image Data

**Hannes Köhler**1**, Annekatrin Pfahl**1**, Yusef Moulla**^2^**, Madeleine Thomaßen**^2^**, Marianne Maktabi**1**, Ines Gockel**2 **Thomas Neumuth**1**, Andreas Melzer**1**, Claire Chalopin**1

1Innovation Center Computer Assisted Surgery (ICCAS), Faculty of Medicine, Leipzig University, Leipzig, 04103, Germany

2Department of Visceral, Thoracic, Transplant, and Vascular Surgery, University Hospital of Leipzig, Leipzig, 04103, Germany

**Supplementary Data S1: Raw data**

**Description:** Video data (video_38369-39119.avi) and related ground truth annotations (annotations.xml) are bundled in file S1.zip for direct import in the open-source Computer Vision Annotation Tool (CVAT).

**Supplementary Video S2: Ground truth annotations on video**

**Description:** Manually annotated landmarks are drawn on each frame of video_38369-39119.avi and shown in video S2.avi. Visible landmarks of the current frame are drawn as green and occluded landmarks as black crosses.

**Supplementary Figure S3: Processing pipeline**

**Description:** The processing pipeline is illustrated as a flow chart in S3.pdf. The four main steps are:

1. Manual registration of the two image sensors (only once)
2. Preprocessing, keypoint detection, and matching in masked color images
3. Calculation of transformation matrices from point pairs
4. Overlay of static HSI data on the current video frame (illustrated as the colorized frame t0)

**Supplementary Video S4: Image registration and overlay**

**Description:** Registration of start frame 20 with the subsequent frames based on SH and A-KAZE is shown S4.avi. The position of the ground truth (green) and the transformed (blue) points are drawn on the semitransparent overlay of the aligned images. No quality check was performed.

**Supplementary Figure S5: Reprojection error of single markers**

**Description:** Reprojection error (RE), moved distance, and normed RE for each annotated marker of start frame 20 are given as mean and standard deviation over all consecutive frames with more than 15 corresponding manual annotations. The homography for image transformation was obtained with ORB1000. For detailed information, the RE and normed RE of a good (11), medium (10), and poor (20) marker are shown for each frame. Three exemplary frames (60, 380, and 450) illustrate the ground truth position of the markers (green) and the position obtained after image transformation (blue). Non-overlapping regions are shown in greyscale.
